# Interference with Activator Protein-2 transcription factors leads to induction of apoptosis and an increase in chemo- and radiation-sensitivity in breast cancer cells

**DOI:** 10.1186/1471-2407-10-192

**Published:** 2010-05-11

**Authors:** Verena Thewes, Francesca Orso, Richard Jäger, Dawid Eckert, Sabine Schäfer, Gregor Kirfel, Stephan Garbe, Daniela Taverna, Hubert Schorle

**Affiliations:** 1 Department of Developmental Pathology, Institute of Pathology, University of Bonn, Medical School, Germany; 2 Molecular Biotechnology Center (MBC) and Department of Oncological Sciences, University of Turin, Via Nizza, 52, 10126 Torino, Italy; 3 Center for Complex Systems in Molecular Biology and Medicine, University of Turin, Via Acc. Albertina, 13, 10023 Torino, Italy; 4 Department of Cell Biology, University of Bonn, Germany; 5 Department of Radiology, University of Bonn, Medical School, Germany; 6 Division Molecular Genetics, German Cancer Research Center (DKFZ), Heidelberg, Germany; 7 National Centre for Biomedical Engineering Science, National University of Ireland, Galway, Ireland

## Abstract

**Background:**

Activator Protein-2 (AP-2) transcription factors are critically involved in a variety of fundamental cellular processes such as proliferation, differentiation and apoptosis and have also been implicated in carcinogenesis. Expression of the family members AP-2α and AP-2γ is particularly well documented in malignancies of the female breast. Despite increasing evaluation of single AP-2 isoforms in mammary tumors the functional role of concerted expression of multiple AP-2 isoforms in breast cancer remains to be elucidated. AP-2 proteins can form homo- or heterodimers, and there is growing evidence that the net effect whether a cell will proliferate, undergo apoptosis or differentiate is partly dependent on the balance between different AP-2 isoforms.

**Methods:**

We simultaneously interfered with all AP-2 isoforms expressed in ErbB-2-positive murine N202.1A breast cancer cells by conditionally over-expressing a dominant-negative AP-2 mutant.

**Results:**

We show that interference with AP-2 protein function lead to reduced cell number, induced apoptosis and increased chemo- and radiation-sensitivity. Analysis of global gene expression changes upon interference with AP-2 proteins identified 139 modulated genes (90 up-regulated, 49 down-regulated) compared with control cells. Gene Ontology (GO) investigations for these genes revealed *Cell Death *and *Cell Adhesion and Migration *as the main functional categories including 25 and 12 genes, respectively. By using information obtained from Ingenuity Pathway Analysis Systems we were able to present proven or potential connections between AP-2 regulated genes involved in cell death and response to chemo- and radiation therapy, (i.e. *Ctgf, Nrp1*, *Tnfaip3, Gsta3*) and AP-2 and other main apoptosis players and to create a unique network.

**Conclusions:**

Expression of AP-2 transcription factors in breast cancer cells supports proliferation and contributes to chemo- and radiation-resistance of tumor cells by impairing the ability to induce apoptosis. Therefore, interference with AP-2 function could increase the sensitivity of tumor cells towards therapeutic intervention.

## Background

The familiy of Activator Proteins-2 (AP-2, Tcfap2) comprises 5 highly conserved DNA-binding transcription factors referred to as AP-2α, AP-2β, AP-2γ, AP-2δ and AP-2ε (or Tcfap2a-e) [1]. They preferentially bind GC-rich consensus-sequences in their target genes, which results in transcriptional regulation either as a stimulatory or repressive event [1,2]. All AP-2 proteins share a modular protein structure consisting of a proline/glutamine-rich transactivation domain at the amino terminus, followed by a highly conserved central basic region and a helix-span-helix motif at the carboxyl terminus. DNA-binding is mediated by the basic region and requires dimer formation of two AP-2 proteins via the helix-span-helix motifs. AP-2 proteins are involved in manifold cellular functions such as proliferation, differentiation and apoptosis and play pivotal roles during embryonic development and carcinogenesis [1]. Especially the family members AP-2α and AP-2γ have been implicated in breast cancer [3]. Under physiological conditions expression of AP-2α and AP-2γ is restricted to either the luminal or the myoepithelial compartment in the breast, respectively [4,5]. In contrast simultaneous overexpression of AP-2α and AP-2γ has been observed in breast carcinoma [4], but their impact on mammary tumorigenesis is still discussed controversially [6].

Several studies propose a tumor-suppressive function for AP-2α in breast tissue [7-9] and data from clinical trials indicate that tumor progression is accompanied by loss of AP-2α expression [10]. Moreover, loss of heterozygosity on chromosome position 6p22, where AP-2α is mapped to, is frequently observed in breast cancer specimens [11]. Accordingly, high expression of AP-2α in invasive breast cancer is correlated with favorable overall survival rates in patients [4].

Furthermore, in transgenic mouse studies, overexpression of AP-2α in the mammary epithelium resulted in impaired mammary gland growth caused by a reduction in proliferation and a simultaneous increase in apoptosis [12]. This fits *in vitro *data where loss of AP-2α lead to a decrease of apoptosis and an increased resistance towards chemotherapeutic drugs [8]. These effects might be partly mediated through control of expression of the CdK-Inhibitor p21^WAF ^and of p53 by AP-2α, which supports the idea that AP-2α acts as a tumor suppressor [9,13,14].

In contrast, little is known about the actual role of AP-2γ in breast carcinogenesis. Overexpression of AP-2γ is frequently seen in breast tumors and breast cancer cell lines [15,16] and has recently been shown to correlate with poorer response to hormone therapy and reduced patient survival in invasive breast cancer [17,18]. In addition, increased expression of AP-2γ has been associated with poorly differentiated breast tumor samples [4]. Gene amplification has been proposed as potential mechanism leading to overexpression of AP-2γ because the gene maps to the genomic locus 20q13.2, which is frequently amplified in breast cancer [19]. Transgenic mouse studies provide further evidence for a role of AP-2γ in breast tumor formation: MMTV-driven overexpression of AP-2γ in the mammary epithelium leads to increase in proliferation and an impaired differentiation [20]. Moreover, bi-transgenic mice expressing the oncogenic HER-2/neu receptor tyrosine kinase and AP-2γ showed an accelerated tumor progression compared to single-transgenic HER-2/neu mice [21]. Together, these findings point towards a causal involvement of AP-2γ in the etiology of breast cancer.

AP-2 proteins form either homo- or heterodimers [1]. There is evidence that under physiological conditions the net effect whether a cell proliferates, undergoes apoptosis or differentiates, may depend on the balance between different AP-2 isoforms [22]. So far, experiments addressing AP-2 functions in breast cancer cells were using either overexpression of, or interference with particular AP-2 isoforms. Overexpression of transcription factors may lead to occupancy of promoters not used under physiological conditions, which masks specific AP-2 functions. In the latter case, interference with a particular isoform may cause a shift in AP-2 dimer composition and hence obscure the physiological function.

In a complementary approach, we now set out to investigate the role of AP-2 proteins in a global manner by interfering with them functionally. To this end, we conditionally overexpressed a dominant-negative mutant (ΔAP-2γ) in the N202.1A murine breast cancer cell line expressing AP-2α and AP-2γ endogenously. Interference with AP-2 proteins influenced the expression of genes involved in chemo- and radiation-sensitivity and resulted in the induction of apoptosis. These results establish an important role for AP-2 transcription factors in breast cancer cells determining their sensitivity towards chemotherapeutic agents and ionizing radiation. Expression of AP-2 proteins in breast tumors might therefore be of prognostic value. The results also suggest that AP-2 proteins or their target genes may be promising targets for therapeutic intervention in breast cancer.

## Methods

### Plasmids

pUHG172-1neo (rtTA) was kindly provided by H. Bujard (Heidelberg, Germany). In order to yield pBIEGFP, EGFP was cloned from pEGFP-C3 into pBI-4 (kindly provided by H. Bujard (Heidelberg, Germany) as an NheI/XbaI fragment. ΔAP-2γ was created by deleting 474 bp (158 aa of the NH-terminus) of the 5'-portion of the murine AP-2γ cDNA. Upon introduction of a start codon using a NotI/NaeI-flanked oligo, ΔAP-2γ was cloned into pBIEGFP using NotI/Sal I restriction sites to generate pBIEGFPΔAP-2γ. The BMP-4 Firefly luciferase construct was kindly provided by M. Moser, (Martinsried, Germany) and the CMV-Renilla luciferase plasmid was obtained from Promega (Mannheim, Germany).

### Cell Lines and Cell Culture

The murine breast cancer cell line N202.1A is derived from MMTV-HER-2/neu transgenic mice and was kindly provided by P.-L. Lollini (Bologna, Italy) [22]. N202.1A cells were grown in DMEM-Glutamax supplemented with 20% fetal calf serum and penicillin/streptomycin (Invitrogen, Karlsruhe, Germany). Cells were kept under standard conditions using a cell culture type incubator at 37°C under 7.5% CO_2_. Functional assays were performed 96 h after addition of doxycycline to the cell culture media (2 μg/ml, BD Biosciences, Heidelberg, Germany).

### Transient and stable Transfection

For stable transfection N202.1A cells were transfected using 39 μg plasmid DNA (ratio plasmid - resistance gene 10: 1) using 107.5 μl Lipofectamine 2000 (Invitrogen, Karlsruhe, Germany) per 10 cm culture dish according to the manufacturer's protocol. N202.1A cells were selected at 850 μg/ml G418 (Calbiochem, Darmstadt, Germany) for the pUHG172-1neo and 200 μg/ml Hygromycin B (BD Biosciences, Heidelberg, Germany) for pBIEGFPΔAP-2γ and pBIEGFP. For BMP-4 luciferase assays N202.1A cells were transfected with 900 ng plasmid (870 ng BMP-4 Firefly luc +30 ng CMV-Renilla luc) and 1.75 μl Lipofectamine 2000 (Invitrogen, Mannheim, Germany) per 24 well culture dish according to the manufacturer's protocol.

### Microscopy and Image Processing

Cells were visualized using a Leica-DM-IRB microscope (Bensheim, Germany), fitted to a Microfire digital camera (Optronics, Goleta, CA, USA) and image processing was performed applying Adobe Photoshop and Illustrator software.

### Co-Immunoprecipitation

HCT116 cells were transfected with equal amounts of AP-2α- and ΔAP-2γ - expression plasmids. Cells were lysed after 48 hours with non-denaturing lysis buffer. Co-IP was performed with 20 μl DYNABEADS^®^(Invitrogen Cat.no. 199.03D, Invitrogen, Karlsruhe, Germany) und 1,5 μg anti-AP-2α antibody (H79, Santa Cruz, Heidelberg, Germany). 150 μg protein lysate per sample was loaded. Western blot with anti-AP-2γ antibody (6E4/4, Santa Cruz, Heidelberg, Germany) followed.

### Western Blot Analysis, Luciferase Assays and Giemsa Staining

Western Blot was performed using the following primary antibodies: AP-2γ (6E4/4, 1:200 Upstate, New York, USA), AP-2α (H79, 1:200 Santa Cruz Heidelberg, Germany), AP-2β (1:1000), AP-2δ (1:1500) and AP-2ε (1:1500), all kindly provided by M. Moser (Martinsried, Germany). The following secondary antibodies were used: goat anti-rabbit-HRP (1:2000 DAKO, Hamburg, Germany) and rabbit anti-mouse HRP (1:1000, DAKO, Hamburg, Germany). Protein lysates from HeLa (cervical carcinoma) N2A (neuroblastoma) cell lines and human keratinocytes or *in vitro *synthesized proteins, respectively, served as antibody positive controls for the different AP-2 isoforms in Western Blot analysis. BMP-4 promoter luciferase assays were performed using the "Dual Luciferase Reporter Assay Systems Kit" (Promega, Mannheim, Germany) according to the manufacturer's protocol. 48 h upon induction (2 μg/ml doxycycline), N202.1A cells were transfected with BMP-4-Firefly luc, lysed, and luciferase activity was measured at 562 nm (Berthold Technologies, Bad Wildbad, Germany). Transfection efficiencies were normalized using a CMV-driven Renilla Luciferase (CMV-Renilla luc). To quantifiy cell numbers after treatment with chemotherapeutic compounds and irradiation cells were stained with Giemsa. For this purpose cells were washed twice in ice-cold PBS and fixed in methanol for 5 min at room temperature, stained for 5 min in Giemsa solution (Merck, Darmstadt, Germany) and washed gently in H_2_O. Cells were quantified using ImageJ software (Adriamycin treatment) or three independent fields of visions were counted (irradiation) in each experiment.

### Proliferation

Determination of S-phase index was carried out using the "Click-iT™ EdU Imaging Kit" (Invitrogen, Karlsruhe, Germany) according to the manufacturer's instructions. Briefly, 96 h after addition of doxycycline (2 μg/ml) N202.1A cells were incubated with 100 μM thymidine analogon EdU for 30 min at 37°C, fixed in 3.7% formaldehyde for 15 min and washed twice in 3% BSA/PBS. Permeabilization was achieved using 0.5% Triton X-100/PBS for 20 min and cells were washed twice with 3% BSA/PBS. Subsequently, the fixed cells were incubated with the reaction cocktail containing Alexa Fluor^®^594-azide for 30 min and washed with 3% BSA/PBS. Imaging using a Leica DM-IRB (Leica, Bensheim, Germany) microscope was followed by quantification of Edu Alexa Fluor^®^594-azide staining in three fields of vision (magnification ×100) in three independent experiments. On average, a field of vision contained 218 cells.

### Transmission (TEM) - and scanning electron microscopy (SEM)

For TEM-analysis, cells were fixed in 1.25% glutaralaldehyde in 0.1 M cacodylate buffer (pH 7.4) for 1 h and post-fixed in 2% OsO_4 _in cacodylate. Following dehydration in a graded series of ethanol, the specimens were embedded in a propylene-epon-mixture, mounted as 50 nm thin sections and post-contrasted with 3.5% uranylacetate. TEM analysis was performed using a CM10 microscope (Philips, Eindhoven, Netherland).

For SEM cells were fixed in 2% glutaralaldehyde in 0.1 M sodium cacodylate (pH 7.3) for 20 min, transferred to 0.1% aqueous tannic acid and rinsed with distilled water. All specimens were dehydrated through a graded series of ethanol and critical point-dried from CO_2 _in 10 cycles using a Balzers CPD 030 (BAL-TEC, Schalksmuehlen, Germany). Dried specimens were mounted on aluminium sample holders and coated with a 2 nm layer of platinum/palladium in a HE 208 sputter coating device (Cressington, Watford, UK). SEM analyis was performed with an XL 30 SFEG (Philips, Eindhoven, Netherlands).

### Caspase 3/7 Acitivity and AnnexinVC3.18 staining

Caspase 3/7 activity was quantified using the "Caspase-Glo^® ^3/7 Assay Kit" (Promega, Mannheim, Germany) according to manufacturer's instructions. Non-induced or cells induced with doxycycline (2 μg/ml) for 96 h were incubated with "Caspase-Glo^® ^3/7 Reagent" for 3 h followed by measurement of luminescence at 562 nm (Berthold Technologies, Bad Wildbad, Germany). AnnexinVC3.18 staining was carried out using the "AnnexinVC.18 Kit" (Sigma-Aldrich, Munich, Germany). Doxycycline-induced cells were washed 3 times in PBS followed by 3 cycles of binding buffer for 1 min. In the next step cells were incubated with the AnnexinVC3.18 conjugate (AnnexinVC3.18:binding buffer, 1:100) for 10 min at room temperature, washed 3 times in binding buffer and then subjected to fluorescence microscopy. Cells treated for 2.5 h with Staurosporine (2 μM) served as positive controls (Sigma-Aldrich, Munich, Germany).

### Irradiation

The irradiation of the N202.1 cells 96 h after addition of doxycyline (2 μg/ml) at 105 Gy was performed using a linear accelerator (Siemens Mevaton MD2, Siemens Medizintechnik, Munich, Germany). The photon energy of 6 MeV at a dose rate of 2 Gy/min was chosen. The field size was set to 20 cm × 20 cm at a SSD (skin to surface distance) of 100 cm. The beam divergence was approx. 11°. The cells were irradiated in a 6-well plate using a RW3-Phantom (PTW) at dose maximum. The photon beam was calibrated according the DIN 6800-2 protocol for a water equivalent energy dose. The RBW-factor for 6 MeV irradiation is almost 1, so the energy dose was equivalent to the biological dose.

### RNA isolation and qRT-PCR

Total RNA was isolated from the various clones using Trizol Reagent (Invitrogen, Karlsruhe, Germany) according to the manufacturer's instructions. 1 μg of DNase-treated (DNA-free™ kit, Ambion, Austin, TX) total RNA was reverse transcribed using RETROscript™ reagents (Ambion, Austin, TX). RNA was heat-denatured for 3' at 85°C and the reaction was incubated at 42°C for 1 hour and 10' at 92°C. Quantitative Real Time PCR (qRT-PCR) reactions were carried out in 96 well plates using SYBRGreen^®^Master Mix (Applied Biosystems, Foster City, CA), specific primers and 10 ng total RNA converted into cDNA in 10 μl final volume. Fluorescence was measured using an ABI Prism^® ^7300 (Applied Biosystems, Foster City, CA) detection system according to the manufacturer's instructions. Primers were purchased from QIAGEN (QuantiTect^® ^Primer Assays). Relative quantitations to control cells were performed: first, each Ct value was corrected for the Ct_r _of the reference gene, GAPDH, and then the Ct of each sample was subtracted from the Ct of control cells (Ct_0)_. The relative amount of template (Q) was therefore calculated as: . All samples were run in triplicates and mean and standard deviation calculated as described in Bookout et al. 2003 [23]. QuantiTect^® ^Primer Assay catalogue numbers are as follows: QT00101297 Mm_Tcfap2c_1_SG QuantiTect Primer Assay (200) (NM_009335, NM_001159696); QT00265524 Mm_Egr3_1_SG QuantiTect Primer Assay (200) (NM_018781); QT00096131 Mm_Ctgf_1_SG QuantiTect Primer Assay (200) (NM_010217); QT01044295 Mm_Sema3b_1_SG QuantiTect Primer Assay (200) (NM_009153); QT00157381 Mm_Nrp1_1_SG QuantiTect Primer Assay (200) (NM_008737); QT01061599 Mm_Gsta3_1_SG QuantiTect Primer Assay (200) (NM_010356); QT00100653 Mm_Gzme_1_SG QuantiTect Primer Assay (200) (NM_010373); QT00134064 Mm_Tnfaip3_1_SG QuantiTect Primer Assay (200) (NM_009397). QT00105483 Mm_Fst_1_SG QuantiTect Primer Assay (200) (NM_008046).

### Northern Blot

Northern Blot analysis was carried out as described in Jäger et al. [20] using the murine AP-2γ cDNA as a probe.

### Whole genome expression analysis

Microarray analysis of gene expression in response to expression of ΔAP-2γ/EGFP (Δ#7 + Δ#15) or EGFP (Δ#5 + Δ#11) in N202.1A breast cancer clones (2 μg/ml doxycyline for 96 h) was performed using the Illumina BeadChip system (Illumina, Inc, San Diego, CA). We used 500 ng of total RNA to obtain labeled, amplified cRNA for each sample to hybridize the Illumina Ref-8 BeadChips according to manufacturer's instructions (Illumina, Inc, San Diego, CA). Arrays were scanned with an IlluminaBeadArray Reader confocal scanner and data processed and analyzed using IlluminaBeadStudio software (Illumina, Inc, San Diego, CA). Raw Illumina data were rank invariant normalized with BeadStudio software (Illumina, Inc, San Diego, CA), which was also used to assess differential expression between the ΔAP-2γ and control clones, based on three RNA preparations from each clone after subtraction of the background obtained with control clones following doxycyclin treatment. After normalization, genes were filtered by their 'detection' value, which had to be 0.99 (significantly detected), in the three samples. Subsequently, we identified differentially expressed genes using the Illumina custom error model implemented in BeadStudio, which provides an expression difference score ('DiffScore') taking into account background noise and sample variability [24]. We chose a DiffScore threshold of 30, corresponding to a p value of 0.001, with a False Discovery Rate (FDR) lower than 5%. To restrict the analysis to the most regulated genes, an additional filtering criterion was that the average expression fold-change between ΔAP-2γ/EGFP- (Δ#7 or Δ#15) and EGFP- (Δ#5 or Δ#11) expressing clones had to be at least 1.5-fold, which lead to the identification of 139 modulated transcripts (49 decreased and 90 increased). Sample permutation analysis confirmed that under these conditions the FDR was well below 5%.

### Gene Set Enrichment Analysis

Gene Set Enrichment Analysis (GSEA) is a computational method used to look for overlaps between the AP-2-driven gene set obtained from the microarray analysis and modulated genes present in the Molecular Signature Database (MSigDB) following chemical and genetic perturbations [25].

### Chromatin immunoprecipitation (ChIP) assays

ChIP was performed using the ChIP-IT™ Kit (Active Motif, Carlsbad, CA) reagents and protocols. Primer pairs were designed on the TFAP2 binding site containing regions (identified by TRANSFAC) using the Primer-BLAST software http://www.ncbi.nlm.nih.gov/tools/primer-blast/index.cgi?LINK_LOC=NcbiHomeAd. The following primers were used: Tnfaip3FW: 5'-CCCCTAACGGAGGCACTCTTCCAC-3'; Tnfaip3RV: 5'-CCGCCTCCTCCAGGTCTTCCTAGCCC-3'; CtgfFW: 5'-AGGAAGTCTC GGGCCTCTTCTCTTTGA-3';CtgfRV: 5'-TCAAGTGGCTGACCACATCATCTGCAC-3'.

PCR was performed using Platinum^® ^*Taq *DNA Polymerase according to the manufacturer's instructions.

## Results

### Establishment of N202.1A breast cancer cells conditionally expressing ΔAP-2γ using the Tet-ON^® ^System

**Figure 1:**
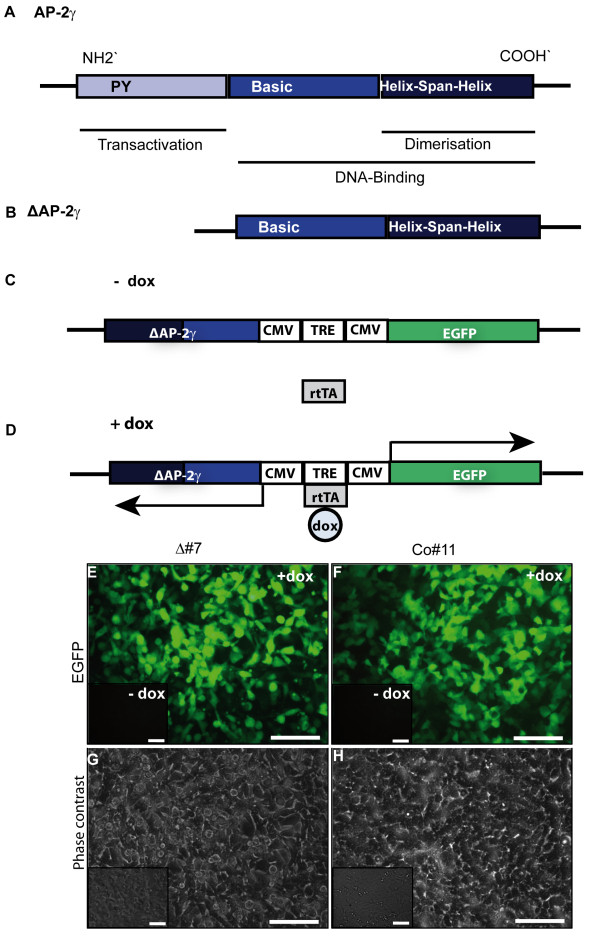
**Establishment of the conditional ΔAP-2γ expression system using the Tet-ON^® ^System in N202.1A breast cancer cells**. (A) The schematic representation of the structural AP-2 protein domains shows the N'-terminal proline- and glutamine-rich (PY) transactivation domain, the DNA-binding domain which consists of the basic region and the helix-span-helix motif and the C'-terminal dimerization domain. (B) The dominant-negative AP-2γ mutant (ΔAP-2γ) has a deleted transactivation domain and therefore an abolished transactivation potential. It still dimerizes with full length AP-2 proteins, thereby inhibiting their function. (C, D) Usage of a bidirectional Tet Responsive Element promoter (TRE) allowed for conditional coexpression of ΔAP-2γ and EGFP upon addition of doxycycline (+dox, 2 μg/ml) in rtTA containing N202.1A cells. (E-H) Generation of N202.1A clones expressing ΔAP-2γ and/or EGFP respectively: stably transfected N202.1A rtTA breast cancer cells were screened for conditional expression of rtTA and either ΔAP-2γ and EGFP (E, Δ#7) or EGFP only (F, Co#11) using fluorescence (E, F) and phase contrast (G, H) microscopy. Clones display a low backround expression in the uninduced state (-dox) but high transgene expression upon induction (+dox, 2 μg/ml). Scale Bar in E-H represents 50 μm.

To assess the role of AP-2 proteins in breast cancer we took advantage of the murine N202.1A breast cancer cell line [26], which had been derived from a mammary tumor of a MMTV-HER-2/neu transgenic mouse and expresses AP-2α and AP-2γ. To functionally interfere with AP-2 proteins, we constructed a dominant-negative mutant of the transcription factor AP-2 (Figure 1A). For this purpose we used a truncated cDNA of the murine AP-2γ lacking the 5'-portion, which encodes the 158 amino acids of the transactivation domain. This mutant, referred to as ΔAP-2γ (Figure 1B), is still able to dimerize but fails to regulate target genes due to the lack of its transactivation domain. It therefore interferes with AP-2 function in a dominant-negative manner [27,28]. Conditional expression of the ΔAP-2γ construct was achieved using the Tet-ON^® ^System with a bidirectional promoter allowing for simultaneous expression of the dominant-negative AP-2γ molecule (ΔAP-2γ) and the reporter Enhanced Green Fluorescent Protein (EGFP, Figure 1C) in a doxycycline-dependent manner (Figure 1D). Stable transfection of N202.1A cells with these constructs yielded clones conditionally coexpressing the ΔAP-2γ and EGFP upon addition of doxycycline (Figure 1E, G compare to inset). To exclude clonal effects due to different copy numbers or integration sites of the transfected constructs all analyses were performed with two independently derived clones (Δ#7, Δ#15). Furthermore we created control clones, conditionally expressing only EGFP (Co#5, Co#11), in order to exclude unspecific effects caused by EGFP expression or by doxycycline treatment (Figure 1F, H, compare to inset).

**Figure 2:**
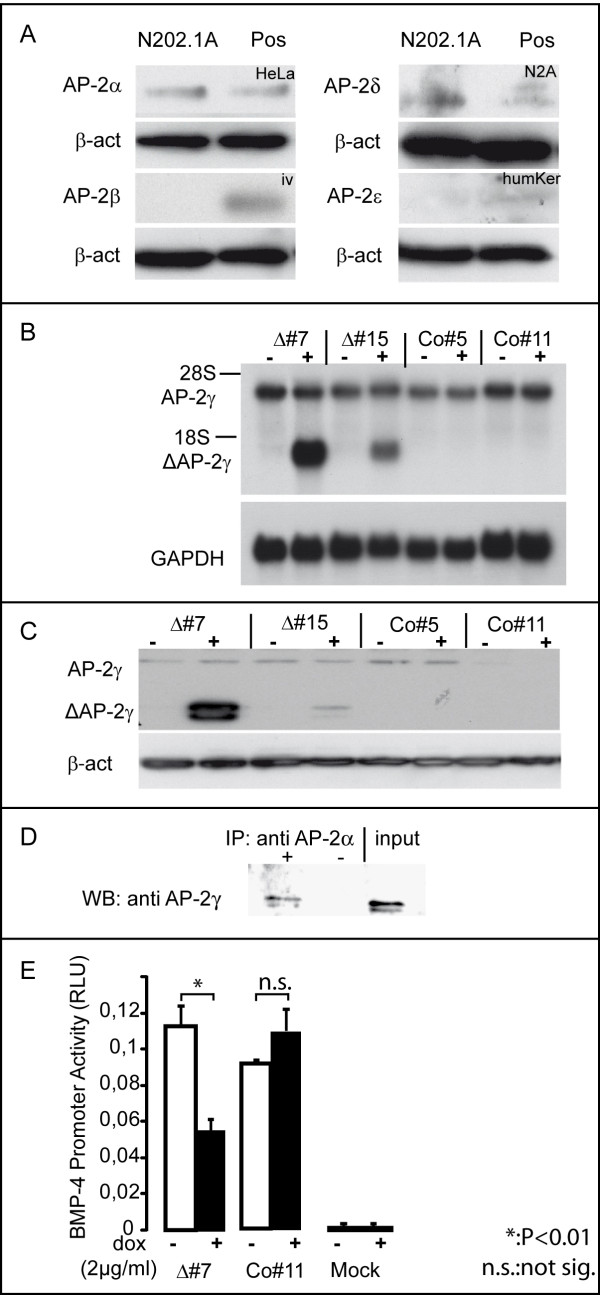
**AP-2 isoform and conditional ΔAP-2γ expression in N202.1A cells. Verification of ΔAP-2γ function using BMP-4 luciferase promoter assays**. (A) Western-blot using antibodies detecting the different AP-2 isoforms in N202.1A cells. As positive control (Pos) for the antibodies, we used protein lysate from HeLa cells for AP-2α (HeLa), N2A cells for AP-2δ (N2A), human Keratinocytes for AP-2ε (hum Ker) and in vitro translated protein for AP-2β (iv). (B) Northern blot analysis of ΔAP-2γ expression in the stable N202.1A clones, uninduced or induced with doxycycline (2 μg/ml). Fifteen micrograms of total RNA were resolved on a formaldehyde gel, transferred to a Nylon membrane and hybridized with a P-32-labelled AP-2γ cDNA probe. GAPDH probe was used to monitor the amounts of RNA. (C) N202.1A cells were induced with doxycycline (2 μg/ml) for 96h and subjected to western blot analysis. Δ#7 and Δ#15 show strong transgene expression in a doxycyline dependent-manner (32 kDa), which is not detectable in Co#5 and Co#11. Of note, the antibody also detects endogeneous AP-2γ (50 kDa) which is not affected by doxycycline. (D) HCT116 cells were transfected with expression constructs for AP-2α and ΔAP-2γ. Co-IP experiment using antibody to AP-2γ for IP and antibody to AP-2α to detect heterodimerization between ΔAP-2γ and AP-2α. - no Antibody; + IP using AP-2γ Antibody; input control. (E) For BMP-4-promoter luciferase assays N202.1A cells were induced with doxycycline for 96 h and transfected with BMP-4 luciferase. 48 h after transfection luciferase activity was quantified. For internal normalization of transfection efficiency a CMV-driven renilla luciferase was used.

Western blot analyses demonstrated the expression of AP-2α and little amounts AP-2δ in N202.1A cells (Figure 2A). Subsequent northern- and western blot analyses showed expression of AP-2γ in the cells and further documented, that the clones strongly over-express ΔAP-2γ both on RNA and on protein level after addition of doxycycline. Of note, no band indicative of expression of ΔAP-2γ could be detected in the absence of doxycline. Also, expression of ΔAP-2γ did not affect the level of endogenous AP-2γ (Figure 2B, C). Using CoIP we demonstrated the ability of the truncated ΔAP-2γ molecule to heterodimerize with AP-2α as well (Figure 2D). To confirm the functionality of ΔAP-2γ, we performed a luciferase reporter assay using a BMP-4 promoter element. BMP-4 expression is modulated by AP-2 transcription factors (unpublished data). The expression of ΔAP-2γ significantly reduced the luciferase signal in the ΔAP-2γ (P < 0.01) clones but not in controls (Figure 2E). Therefore we have established a system which allows for functional interference with AP-2 transcription factors in N202.1A mammary tumor cells.

### Whole genome expression analysis and target gene identification

**Figure 3:**
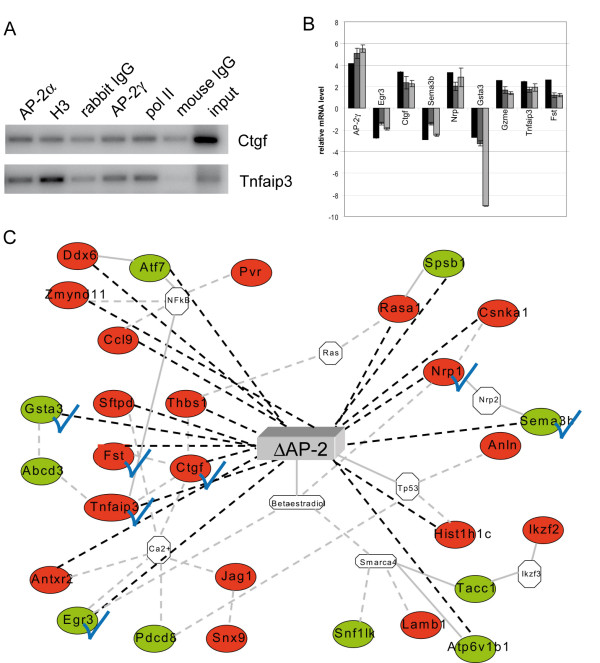
**Microarray analysis validation and functional network for ΔAP-2γ/EGFP- expressing N202.1A breast cancer cells**. (A) Chromatin from N202.1a cells was cross-linked to proteins, extracted and immunoprecipitated with either AP-2α (H-79) or AP-2γ (6E/4) Abs or non-specific rabbit- or mouse-IgG (negative isotype controls) or H3-histone or RNA-polymerase II Abs (positive controls). DNA was analyzed by PCR, using primers flanking the AP-2 putative binding sites in *Ctgf *and *Tnfaip3 *promoters. Input: non immunoprecipitated DNA. (B) Microarray data (additional file 1: Table S1 and Table 1) were validated by qRT-PCR performed in triplicate for 9 genes on three different RNA preparations from ΔAP-2γ/EGFP- (Δ#7; Δ#15) or EGFP- (Co#5; Co#11) expressing N202.1A clones. Black bars: microarray results; Dark grey bars: qRT-PCR of Δ#7 and Δ#15 clones versus Co#5 and Co#11 clones; Light grey bars: qRT-PCR Δ#7 clone versus Co#11 clone. The GAPDH gene was used as an internal control to normalize the data. Microarray analysis and qRT-PCR-fold changes are shown for each validated gene as average values. Bars represent ± standard error. (C) Functional network which connects the identified AP-2-regulated genes involved in "cell death" taken in part from analyses carried on with Ingenuity™ Pathway Analysis. Legend: Continuous grey lines indicate direct interactions experimentally proven; dashed grey lines represent potential indirect connections; dashed black lines represent potential indirect connections obtained from our microarray results considering only Fold Changes > 2. The green and red symbols represent down- and up-regulations, respectively, while the white symbols indicate genes absent in the dataset but related with the microarray genes as indicated from the literature. Blue checkmarks indicate the genes verified by qRT-PCR in (B).

To better understand the role of AP-2 transcription factors in initiation and progression of breast cancer, we set out to identify AP-2 regulated genes using whole genome expression analysis. The global patterns of gene expression were analyzed in ΔAP-2γ expressing (#7 and #15) clones versus control (#5 and #11) clones in presence of doxycycline. In total 139 differentially expressed genes were found. In order to correlate gene expression to promoter occupancy, the promoter regions of 96 genes were analyzed using Transfac analyses to define consensus binding sites for AP-2 proteins. Of these 96 candidate genes, 52 genes displayed AP-2 binding sites indicating a direct regulation (additional file 1: Table S1). Sequence alignments revealed, that 23 of the 52 binding sites are conserved between mouse and human, further suggesting a functional role for AP-2 in regulating these genes. (additional file 1: Table S1, genes marked in red). To investigate whether AP-2 transcription factors directly bind to these target genes '*in vivo*' chromatin immunoprecipitation was performed on promoter elements of *Connective Tissue Growth Factor (Ctgf) *and *Tumor necrosis factor alpha-induced protein 3 (Tnfaip3)*. Both AP-2α and AP-2γ are recruited to promoter regions of these genes, confirming a direct regulation (Figure 3A). Differentially expressed genes were distributed in different Gene Ontology (GO) classes, the main one being cell death (25 genes), followed by cell adhesion and cell movement (12 genes), and nucleosome assembly (8 genes). In particular, several cell death players were found and listed in Table 1. Among them *Connective Tissue Growth Factor (Ctgf)*, *Neuropilin1 (Nrp1), Chemokine C-C motif ligand 9 (Ccl9) *and *Tumor necrosis factor alpha-induced protein 3 (Tnfaip3) *were highly up-regulated suggesting a repressive role of AP-2 on these genes. On the other hand *Semaphorin 3B (Sema3b) *and *Early Growth Response 3 (Egr3) *were strongly down-regulated suggesting an activating function of AP-2 on these genes. Microarray data were validated by qRT-PCR performed on three different RNA preparations for each clone for 6 genes involved in cell death and for 3 genes belonging to other GO classes, all showing a fold change (FC) >2.0 (Figure3B). Differential gene expression obtained from microarray analysis was compared with data resulting from clones Δ#7 and Δ#15 versus Co#5 and Co#11 or from clone Δ#7 versus Co#11. Data were normalized using the GAPDH gene as internal control. Using some of the information obtained with Ingenuity™ Pathway Analysis Systems we were able to build a simplified network for genes involved in cell death (Figure 3C).

**Table 1:** Cell death-related genes in ΔAP-2γ/EGFP- expressingN202.1A cells

Accession	Symbol	Definition	FC
NM_010217	Ctgf	connective tissue growth factor (Ctgf), mRNA.	3,4
NM_008737.1	Nrp	neuropilin (Nrp), mRNA.	3,3
NM_011338	Ccl9	chemokine (C-C motif) ligand 9 (Ccl9), mRNA.	3,3
NM_015786	Hist1h1c	histone 1, H1c (Hist1h1c), mRNA.	3,1
NM_009160.1	Sftpd	surfactant associated protein D (Sftpd), mRNA.	2,9
NM_008046.1	Fst	follistatin (Fst), mRNA.	2,6
AK031617	Csnk2a1-rs3	casein kinase II, alpha 1 related sequence 3, mRNA	2,5
NM_010762.2	Mal	myelin and lymphocyte protein, T-cell differentiation protein (Mal), mRNA.	2,5
NM_009397.2	Tnfaip3	tumor necrosis factor, alpha-induced protein 3 (Tnfaip3), mRNA.	2,5
NM_011580.1	Thbs1	thrombospondin 1 (Thbs1), mRNA.	2,3
NM_144516.1	Zmynd11	zinc finger, MYND domain containing 11, mRNA	2,3
NM_080428	Fbxw7	F-box and WD-40 domain protein 7, archipelago homolog (Drosophila) (Fbxw7), mRNA.	2,2
NM_145452	Rasa1	RAS p21 protein activator 1 (Rasa1), mRNA.	2,1
NM_133853.1	Magi3	membrane associated guanlylate kinase, WW and PDZ domain containig 3	2,0
NM_133738	Antxr2	anthrax toxin receptor 2, mRNA	2,0
NM_028390.1	Anln	anillin, actin binding protein (scraps homolog, Drosophila) (Anln), mRNA.	1,9
NM_012019.2	Pdcd8	programmed cell death 8 (Pdcd8), mRNA.	1,9
NM_013822.2	Jag1	jagged 1 (Jag1), mRNA.	1,8
NM_010789.1	Meis1	myeloid ecotropic viral integration site 1 (Meis1), mRNA.	1,7
J05277.1	Hk1	hexokinase mRNA, complete cds.	-2,0
NM_177089.3	Tacc1	Transforming, acidic coiled coil protein 1	-2,0
NM_010777.1	Mbp	myelin basic protein (Mbp), mRNA.	2,5
XM_483957	Dyrk2	dual-specificity tyrosine-(Y)-phosphorylation regulated kinase 2, mRNA	2,5
NM_018781	Egr3	early growth response 3 (Egr3), mRNA.	-2,7
NM_009153.1	Sema3b	sema domain, immunoglobulin domain (Ig), short basic domain, secreted, (semaphorin) 3B (Sema3b), mRNA.	-2,9

Microarray analysis (Illumina Ref-8 BeadChip) was performed on ΔAP-2γ (Δ#7, Δ#15) or EGFP- (Co#5, Co#11) expressing N202.1A clones in triplicate and 25 modulated genes involved in cell death (6 decreased, 19 increased, see Methods) were found. FC = Fold change. p < 0.001; FC. +/- 1.5.

### Gene Set Enrichment Analysis

By using the computational Gene Set Enrichment Analysis (GSEA) we looked for gene overlaps between our AP-2-dependent differentially expressed genes (additional file 1: Table 1) and a collection of twenty chemical and genetic perturbation gene datasets spanning 16,271 genes present in the Molecular Signature Database (MSigDB, http://www.broadinstitute.org/gsea/msigdb/) [25]. Results are reported in additional file 2: Table 2, where we found that modulation of the AP-2 dependent genes, such as *thrombospondin 1, tumor necrosis factor, alpha-induced protein 3, jagged 1, semaphorin3B, early growth response3, anillin, collagen type X alpha 1, hydroxysteroid(11-beta)dehydrogenase 1 *have also been found in collections of up- or down-regulated genes following chemical and genetic perturbations. These findings reinforce the involvement of AP-2-driven genes with apoptosis and chemo- and radiation-sensitivity.

### Interference with AP-2 proteins increases the chemo- and radiation-sensitivity of N202.1A breast cancer cells

The global gene expression analysis also identified AP-2 regulated genes, which have an impact on sensitivity of cancer cells towards chemotherapeutic drugs and irradiation like for example the *ATP-binding-cassette, subfamilyB (MDR/TAP) member 9 *and *GSTA3 *(Figure 3B) [29,30]. Therefore we tested whether interference with AP-2 proteins has an influence on the chemo- and radiation-sensitivity of N202.1A breast cancer cells.

**Figure 4:**
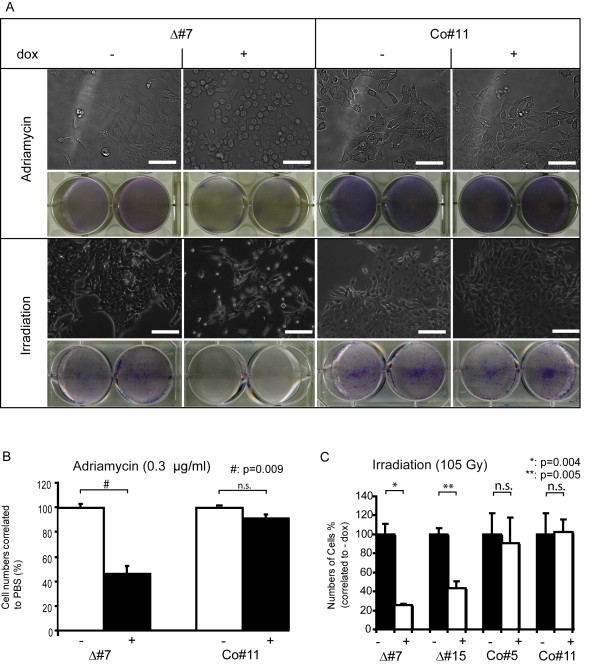
**Interference with AP-2 results in an increase in chemo- and radiation-sensitivity in N202.1A breast cancer cells**. (A) N202.1A cells were left untreated (-) or induced with doxycycline (+) for 96 h and then Adriamycin (0.3 μg/ml) was addded for 72 h (upper columns) or irradiation of 105 Gy was applied (lower columns). Visualisation under the phasecontrast microscope 72 h after treatment with Adriamycin revealed a significant increase in blebbing and dead cells in Δ#7 expressing ΔAP-2γ (+dox) compared to uninduced (-dox) and Co#11 cells (-/+dox). A decreased number of cells is also seen using Giemsa staining upon expression of ΔAP-2γ in Δ#7 (+dox) in comparison to uninduced cells and Co#5 cells. Phase contrast microscopy 72 h after irradiation revealed a higher amount of dead cells in Δ#7 compared to uninduced and Co#11 cells (-/+ dox). Lower amounts of cells are also seen using Giemsa staining upon expression of Δ#7 (+dox) in comparison to uninduced cells (-dox) and Co#11 (-/+dox). Scale Bar represents 50 μm. (B) Quantification of cell numbers shows a decrease in cell numbers of approximately 50% following interference with AP-2 proteins (Δ#7, +dox). Cell numbers are normalized to PBS treated controls. P-value of unpaired *t*-test is given, (n.s. not significant). (C) For determination of radiation-sensitivity the N202.1A clones are induced for 96 h or left uninduced followed by irradiation with 105 Gy. Normalisation of cell numbers to uninduced controls revealed a significant decrease in cell numbers upon expression of ΔAP-2 (Δ#7, +; Δ#15, +). P-value of unpaired *t*-test is given (n.s. not significant).

For this purpose the cells were induced with doxycycline for 96 h and then additionally treated with chemotherapeutic drugs. 72 h after treatment with the topoisomerase II inhibitor Adriamycin, the ΔAP-2γ expressing clones showed an apparent change in morphology; they displayed a more roundish morphology (Figure 4A) compared to the non-induced cells or control cells, which retained a healthy polygonal morphology. After 72 h the cell numbers had dropped to approximately 60% in the cultures where ΔAP-2γ was expressed compared to controls (Figure 4B) (p = 0.009). A similar trend was observed after treatment with Etoposide and Cisplatin (data not shown). These data indicate that repression of AP-2 protein function results in enhanced sensitivity towards chemotherapeutic drugs such as Adriamycin, Etoposide and Cisplatin.

Irradiation represents another main pillar to cure a tumor burden, so we addressed the question whether AP-2 expression impinges on the sensitivity of the cells towards ionizing radiaton. The ΔAP-2γ expressing clones displayed significantly (clone Δ#7: p = 0.0004; clone Δ#15: p= 0.0005) reduced cell numbers 72 h after irrradiation with 105 Gray compared to controls, as revealed by Giemsa-staining (Figure 4A, C). Hence, repression of AP-2 function resulted in increased sensitivity towards ionizing radiation and we conclude that expression of AP-2 genes might confer a selective advantage to breast tumor cells towards chemotherapeutic agents and irradiation.

### Interference with AP-2 proteins causes a reduction in proliferation rate and leads to induction of apoptosis

**Figure 5:**
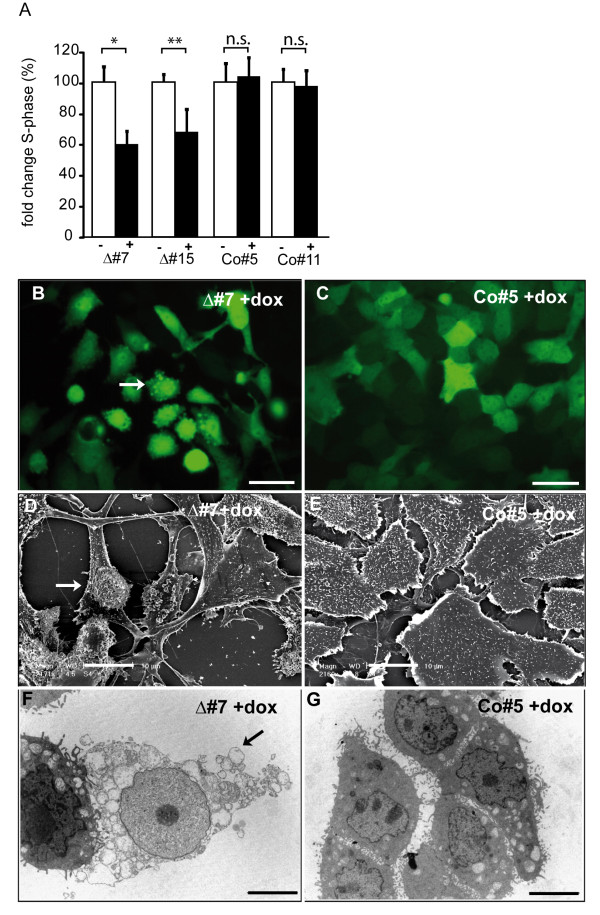
**Impairment of proliferation and enhanced apoptosis upon interference with AP-2 proteins in N202.1A breast cancer cells**. (A) Click-iT Edu assays to determine the amount of cells in S-phase upon expression of ΔAP-2γ in N202.1A. Induced clones were normalized to uninduced clones and the percentage of cells in S-phase determined. *:P < 0.01, **:P < 0.05, n.s.:not significant. (B, C) N202.1A breast tumor cells were treated with doxycycline for 96 h and then subjected to fluorescence, SEM (D, E) and TEM (F, G) analyses. Upon expression of the dominant-negative AP-2γ mutant in N202.1A cells (B, D, F), membrane surface blebbing (indicated by the arrows) can be observed, while control cells display a regular polygonal morphology (C, E, G). Scale Bar represents 20 μm.

To address the question whether interference with AP-2 proteins had an influence on the proliferation rate of N202.1A breast cancer cells, we subjected them to "Click iT™"(Invitrogen) assays which selectively stain cells in S-phase. We detected a decrease in proliferation rate of approximately 30% in clones expressing ΔAP-2γ compared to uninduced und control cells (Figure 5A). These results suggest that AP-2 proteins mediate pro-proliferative functions.

After interference with AP-2 transcription factors we observed changes in morphology of the N202.1A cells. After interference for 96 hrs small protrusions (blebs) on the plasma membrane could be detected (Figure 5B, arrow). These protrusions could be verified using Scanning (Figure 5D) and Transmission (Figure 5F) Electron Microscopy and were not seen in uninduced clones (data not shown) or controls (Figure 5C,E,G). To ascertain if the blebbing was based on changes in the actin cytoskeleton, actin fibers were stained with Phalloidin 96 h after addition of doxycycline. However, we could not detect structural changes of the actin cytosceleton (not shown). This suggests that vesicle formation upon interference with AP-2 function does not involve the modification of actin cytoskeleton structure.

**Figure 6:**
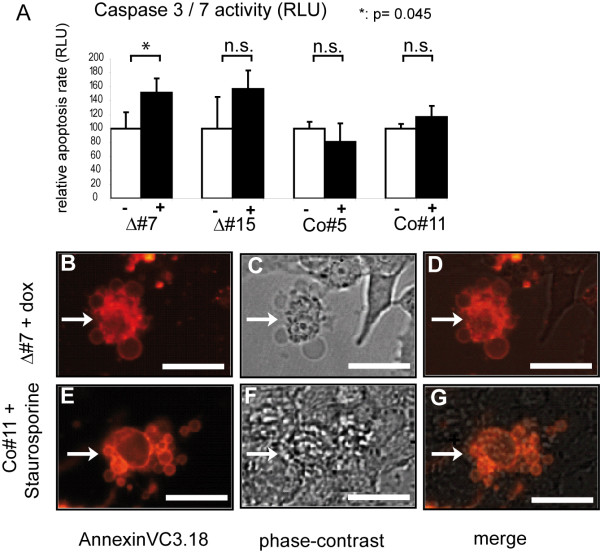
**N202.1A breast cancer cells display membrane-blebbing of upon interference with AP-2 proteins**. (A) N202.1A breast cancer cells were treated with doxycycline for 96 h and Caspase 3/7 activity was quantified using an artificial Caspase 3/7 luminogenic substrate. Induced cells (+dox) expressing the dominant-negative AP-2γ mutant (Δ#7, Δ#15) display a higher Caspase 3/7 activity compared to uninduced cells (-dox) and Co#5, Co#11(-/+dox) cells. Results were derived from three independent experiments each. P-value of unpaired *t*-test is given (n.s. not significant). (B-G) AnnexinVC3.18-staining. N202.1A breast cancer cells are induced for 96 h using doxycycline and then stained with AnnexinV-conjugated Cy3.18 and visualized using fluorescence (B, D, E, G) or phase-contrast optics (C, D, F, G). Cells showing excessive blebbing at the membrane surface following long-term expression of the dominant-negative AP-2 mutant (B-D) are AnnexinV-positive (indicated by the arrows). Control cells treated with Staurosporine as classical inducer of apoptosis morphologically resemble the cells after interference with AP-2 function and are also AnnexinV-positive (E-G) indicating that it is an apoptosis-associated process. Scale Bar represents 10 μm.

We reasoned that the blebs caused by ΔAP-2γ expression might represent apoptotic bodies and therefore checked for other apoptotic markers. First, activity of Caspase 3/7 was measured as an early marker of apoptosis. Untreated clones were compared to doxycycline-treated clones and Caspase 3/7 activity was quantified. Long-time interference with AP-2 function (96 hrs) resulted in a marked increase (p = 0.045 for Δ#7) in Caspase 3/7 activity compared to uninduced and control cells (Figure 6A). Furthermore, a fraction of cells expressing ΔAP-2γ were positive for AnnexinV3.18 staining (Figure 6B-G) which represents another indicator of apoptosis. The morphological changes caused by treatment with the apoptosis-inducer Staurosporine were similar to the blebbing seen upon expression of ΔAP-2γ, suggesting that the ΔAP-2γ expressing cells indeed are undergoing apoptosis (Figure 6E-G, compare to 6B-D).

To summarize our findings, interference with AP-2 transcription factors in N202.1A breast cancer cells resulted in AnnexinV3.18-positive blebbing cells and an increase of Caspase 3/7 acitivity. This further verifies the data from the whole genome expression profiling and indicates that interference with AP-2 proteins sensitizes N202.1A breast cancer cells to apoptosis, which might be one reason for the increase in chemo- and radiation-sensitivity observed upon functional impairment of AP-2 transcriptional activity.

## Discussion

Although single AP-2 proteins have been implicated in tumorigenesis in various types of cancers, their concerted action in initiation and progression of breast cancer is far from being understood. Previous work put its focus on the analysis of the role of single AP-2 isoforms in the etiology of breast cancer. Using a dominant-negative AP-2 mutant, we demonstrated that simultaneous interference with both, AP-2α and AP-2γ leads to a decrease in proliferation and induction of apoptosis in N202.1A breast cancer cells. Furthermore the cells became increasingly sensitive to chemotherapeutic drugs and irradiation.

AP-2α and AP-2γ were suggested to act as decisive pacemakers for cellular fates such as proliferation, apoptosis and differentiation in the mammary epithelium under physiological conditions [5,20]. Therefore, their inappropriate spatio-temporal expression might contribute to malignant transformation. While in the normal breast tissue AP-2α and AP-2γ show non-overlapping expression patterns [5], coexpression of AP-2α and AP-2γ was detected in undifferentiated breast carcinomas [4]. There is increasing evidence that the net outcome whether a cell proliferates, undergoes apoptosis or differentiates depends on the balance of various AP-2 proteins [22]. Here, interference with AP-2 proteins using a dominant-negative AP-2 mutant in N202.1A breast cancer cells resulted in a decreased proliferation rate. Remarkably, overexpression of AP-2γ in the mammary gland using a transgenic mouse approach caused an increase in proliferation as well [20], highlighting the notion that certain AP-2 proteins might trigger proliferation.

Wajapeyee et al. correlated increased AP-2α expression with an enhanced rate of apoptosis after treatment with cytostatic compounds [7,8]. Our results, however, suggest that global interference with AP-2 proteins rather sensitizes cells to apoptosis and anticancer treatment regimen. We obtained conflicting results to those reported for the function of AP-2α alone because we could detect apoptosis and an increased sensitivity towards chemotherapeutic drugs and irradiation after global interference with AP-2 proteins. Our approach benefits from the fact that the dominant-negative AP-2 mutant presumably interferes with all AP-2 homo- and heterodimers present in N202.1A breast cancer cells, which possibly occupy different target gene promoters than AP-2α alone. While this approach can not distinguish between the functions of individual AP-2 family members, it suggests that interfering with AP-2γ may sensitize to apoptosis even in the absence of functional AP-2α.

The whole genome expression analysis revealed 139 differentially expressed genes upon interference with AP-2 proteins. Their function might explain the decrease in proliferation rate and at the same time the increase in chemo- and radiation-sensitivity upon repression of AP-2 function also based on modulations of some of these genes previously found following chemical and genetic perturbations [25]. On mRNA level the following genes were found upregulated upon interference with AP-2 function suggesting that AP-2 proteins act as repressors of these genes: *Connective Tissue Growth Factor (CTGF) *is described to suppress proliferation in breast cancer cells and other tumor entities and induces apoptosis at least in part by activation of caspase-3 [31-33]. Moreover, low expression levels of *CTGF *in breast cancer patients have been correlated with a poorer clinical outcome [34]. *Neuropilin 1 (NRP-1) *receptor has been described to be involved in induction of apoptosis in breast cancer cell lines by inhibiting the Akt-signaling pathway [35,36]. The tumor suppressor gene *Tumor necrosis factor, alpha-induced protein 3 (Tnfaip3) *is an inhibititor of the pro-survival activity of NFκB [37].

Remarkably, the *ATP-binding-cassette, subfamilyB (MDR/TAP) member 9/GSTA3 *was downregulated upon interference with AP-2 function. It is member of a family, which has been shown to contribute to the Multidrug Resistance (MDR) phenotype [30]. Additionally this efflux transporter family has been reported to detoxify reactive oxygen species (ROS) generated by irradiation, [29] which is a potential mechanism how tumor cells acquire resistance towards irradiation-induced damage. This might represent a mechanism of sensitization of N202.1A breast cancer cells towards chemotherapeutic drugs and irradiation caused by the abrogation of AP-2 function.

According to the analysis using ingenuity software, the AP-2 target genes predominantly cluster to NFκB, Tp-53, Ras signalling and Calcium signalling. In summary the concerted deregulation of the candidate genes might render cells more susceptible to apoptosis thus resulting in an increased sensitivity towards chemotherapeutic drugs and irradiation. It remains to be elucidated, whether the candidate genes are direct targets of AP-2 transcription factors or are regulated by an indirect secondary mechanism.

## Conclusions

The simultaneous expression of AP-2 transcription factors in breast cancer cells mediates pro-proliferative and anti-apoptotic functions. In addition they contribute to chemo- and radiation resistance of breast cancer cells. Thus, interference with AP-2 function could increase the sensitivity of tumor cells to therapeutic intervention.

## Competing interests

The authors declare that they have no competing interests.

## Authors' contributions

VT constructed the vectors, generated the cells, performed the induction, radiation and chemotheraphy experiments and writing. FO performed array and ChIP-analyses bioinformatics and interpretation, writing and revision. RJ contributed to conception, acquisition of data, interpretation and writing and performed RNA isolation and northern blots. DE contributed to conception and design, bioinformatics, interpretation, writing and revision of data. GK helped with TEM analyses. SS performed co-immunoprecepitation and western-blot. SG helped with the radiation experiments, DT contributed to interpretation, writing and revision, HS contibuted to conception and design, interpretation, writing and revision. All authors read and approved the final manuscript.

## Pre-publication history

The pre-publication history for this paper can be accessed here:

http://www.biomedcentral.com/1471-2407/10/192/prepub

## Supplementary Material

Additional file 1 **Table S1: Differentially expressed genes in ΔAP-2γ/EGFP-expressing N202.1A breast cancer cells**. Microarray analysis (Illumina Ref-8 BeadChip) was performed on ΔAP-2γ/EGFP- (Δ#7, Δ#15) or EGFP- (Co#5, Co#11) expressing N202.1A clones in triplicate and 139 modulated genes were found (49 decreased, 90 increased, see Methods). FC = Fold change. p < 0.001; FC. +/- 1.5. Functions: more than one Gene Ontology (GO) category was found in some cases. Genes marked in Red display conserved AP-2 binding sites between mouse and human. Numbers and relative location of AP-2 binding site is given (n.d. = not done). Click here for file

Additional file 2 **Table S2: Gene/Gene Set overlap matrix derived by using the datasets deposited in the Molecular Signatures Database**. Row (A) and (B) presents the results of 72 genes identified in the screen here in relation to list all the datasets relevant to the analysis (C-V). The spreadsheet (Overlapgenset) identifies the abbreviations used in (C-V). Red boxes indicate overexpression, green boxes indicate downregulation (Yellow - no information deposited). Click here for file
